# Feasibility of a birth cohort study dedicated to assessing acute infections using symptom diaries and parental collection of biomaterials

**DOI:** 10.1186/s12879-015-1189-0

**Published:** 2015-10-22

**Authors:** Beate Zoch, André Karch, Johannes Dreesman, Masyar Monazahian, Armin Baillot, Rafael T. Mikolajczyk

**Affiliations:** ESME - Research Group Epidemiological and Statistical Methods, Helmholtz Centre for Infection Research, Inhoffenstr. 7, 38124 Braunschweig, Germany; PhD Programme “Epidemiology”, Braunschweig-Hannover, Braunschweig, Germany; German Centre for Infection Research (DZIF), Hannover-Braunschweig site, Braunschweig, Germany; Governmental Institute of Public Health of Lower Saxony, Hannover, Germany; Hannover Medical School (MHH), Hannover, Germany

**Keywords:** Respiratory infections, Gastrointestinal infections, Birth cohort, Symptom diary

## Abstract

**Background:**

A birth cohort dedicated to studying infections in early childhood may be assisted by parental recording of symptoms on a daily basis and a collection of biomaterials. We aimed at testing the feasibility of this approach for use in a long-term study focusing on infections in children in Germany.

**Methods:**

Parents of 1- to 3-year-old children (*n* = 75) were recruited in nursery schools. They were asked to complete a symptom diary on a daily basis and to take monthly and symptom-triggered nasal swabs and stool samples from their child over the study period of three months. Feasibility was measured by means of the return proportions of symptom diaries and bio samples; acceptance was assessed by a questionnaire delivered to participants at the end of the study.

**Results:**

The majority of the participants filled in the symptom diary during the three months study for 75 or more days (77.3 %), and provided the monthly nasal swabs (62.7 %) and stool samples (65.3 %). The time needed for the tasks was acceptable for most participants (symptom diary: 92.3 %, nasal swabs: 98.5 %, stool samples: 100.0 %). In 64.3 % of the symptom-triggered nasal swabs, respiratory viruses were found compared to 55.5 % in throat swabs taken by health-care professionals within the “ARE surveillance Lower Saxony”, a special project by the Governmental Institute of Public Health of Lower Saxony to investigate causal pathogens for acute respiratory infections in children.

**Conclusions:**

The parental assessment of symptoms and collection of biomaterials in a birth cohort dedicated to studying infections appears feasible in a middle class German population. The success of the study will depend on the ability to maintain these activities over a long time period.

**Electronic supplementary material:**

The online version of this article (doi:10.1186/s12879-015-1189-0) contains supplementary material, which is available to authorized users.

## Background

The first years of life represent the most important period for the development of the immune system which is formed by continuous encounters with pathogens as well as symbionts and commensals. Previous birth cohort studies assessed either relations between infectious agents and the immune system, e.g. rhinovirus infection and wheezing/asthma [1, 2]; or between an infants’ gut microbiome and the immune system [3–8]. None of these studies have collected comprehensive information regarding childhood infections and vaccinations, as well as the development of the infants’ microbiome when investigating the development of the immune system and diseases in later life. As more than half of the acute respiratory infections (ARI) and one quarter of the gastrointestinal infections in childhood do not lead to a consultation of a physician [9–13], recording the complete history of infections during childhood can be aided by assistance from the parents in conducting daily observations regarding symptoms and obtaining biomaterials during infection episodes. In a similar fashion, the assessment of changes of the nasal and the gut microbiome over time and in relation to infections and the development of immunity requires regular collection of nasal swabs and stool samples during asymptomatic episodes, which also can be aided by parents’ assistance.

Symptom diaries generally have a long tradition in the assessment of acute infections [14–17] and have been recently used for periods of 1 to 5 years to capture acute respiratory and gastrointestinal infections among children in studies in Australia [10, 11, 18]. Sample collection by health-care professionals to identify respiratory pathogens has been conducted in several studies by asking participants to visit the physician at every infection [19] or by sending study staff to participants every week or every time participants report an infection [11, 20–23]. Just recently, self-collection of nasal swabs [24–28] and of stool samples [29] was tested. Parent-collected nasal swabs have been conducted in previous studies in Australia [10, 18, 30] and Italy [31]. Within the Australian studies [10, 18, 30], parents also collected stool samples from their child. However, in Italy, nasal swabs were taken by parents in a hospital with help on hand if needed [31]. In Australia, samples were taken by parents at home and picked up by study staff from each home upon a phone call of the participants (personal communication S. Lambert). Either approach would be too expensive with respect to both personal and financial resources for a long-term collection of bio samples in a large birth cohort in Germany with the goal of capturing the entire history of infections. If a sample collection was only conducted during hospital visits or consultation of a physician, more than half of the respiratory infections and one quarter of the gastrointestinal infections in childhood would be missed [9–13]. Sending study staff to pick up the samples from the homes of the participants is very resource intensive and not necessary in Germany as, in contrast to Australia, bio samples can be sent by standard mail. However, the additional burden for the parents to pack and send the bio samples has to be considered and tested prior to implementation in a larger study.

The current study assessed the feasibility of a unified approach including symptom diary and parental collection of symptom-triggered as well as regular monthly biomaterials for their use in a future birth cohort. It aimed specifically to:

A) investigate the feasibility of the use of a symptom diary and parent-collected swabs,
B) investigate the acceptance of keeping a symptom diary and obtaining bio samples by parents,
C) analyze predictive factors for compliance with the study protocol over a period of three months,
D) investigate attitudes of parents towards participation in a long-term study with symptom diary, parental bio sampling, and blood obtainment in order to draw conclusions about long-term compliance.

The three-month period was chosen as it was estimated long enough to test the practical feasibility of the data collection approach and the used materials. Since it is not possible to derive implications for long-term compliance from such a short study period, we tried to address this limitation by adding a quantitative survey at the end of the study period in which parents were asked about their attitudes towards a long-term birth cohort study.

We chose the winter/spring period as children are more likely to get sick from respiratory infections in this period, compared to other periods of the year. We were thus able to test the study concept at the time of the parents’ highest workload.

## Methods

### Recruitment

Since in the future this approach should be used in a long-term birth cohort, testing the feasibility of recording symptoms and parental bio sampling appeared most reasonable for children in the age group of 1 to 3 years because these children experience the most infections. The recruitment of parents took place in nursery schools (nurseries) in Braunschweig, Germany, from November 2013 to March 2014. Out of 64 nurseries in Braunschweig taking care of 1- to 3-year-old children, a random sample of 40 was contacted by mail and telephone calls. Three nurseries were allowed to participate as a convenience sample. Flyers and posters were distributed to nurseries, and the study was introduced on-site by study staff during pick up or drop off periods at the nursery.

### Symptom diary and collection of bio samples

Participants were asked to fill in a symptom diary on a daily basis and to obtain several bio samples over a period of three months.

At the beginning of the study period, parents were provided with symptom diaries, materials for the obtainment of the bio samples, instructions on how to obtain the bio samples, and reply-paid envelopes. A trained study member demonstrated obtaining a nasal swab to one of the parents of each study child.

The symptom diary was based on diaries used by Lambert et al. [18] and the Canadian Acute Respiratory Infection Scale (CARIFS) [32, 33] (Additional file 1). Participants could choose to fill in the symptom diary on paper or online. Body temperature was recorded by the parents using their own thermometers without further training. In the analysis, fever was defined as a body temperature above 37.2 °C (axillary), 37.8 °C (oral), or 38 °C (rectal, tympanic, forehead) [34]. Diarrhea was defined as at least three times loose stool in a 24 h period, following the WHO definition [35]. In order to check the validity of the information on visits to a pediatrician provided by the parents in the symptom diary, we asked for permission to contact said pediatricians.

Parents were asked to obtain nasal swabs and stool samples once a month on a fixed day, independently from the occurrence of symptoms, as well as once at the occurrence of symptoms. Parents were asked to rate the behavior of the child during the nasal swabbing on a Likert scale from very calm (value 1) to extremely tense (value 10), as well as their own feelings while obtaining the swab from very confident (value 3) to very unconfident (value 0). Bio samples and short questionnaires were to be mailed as quickly as possible.

The symptom-triggered nasal swabs were tested for a number of viruses using the TaqMan polymerase chain reaction (PCR). Pathogen detection was used as an incentive on the one hand and, on the other hand, as a quality check to test if the sample quality was as good as what can be collected by health-care professionals. Therefore, the virus finding rate was compared to an analysis of 1,326 throat swabs obtained during the same time period by physicians in private practices and hospitals among 1- to 3-year-old children with acute respiratory symptoms. This collection was conducted in the context of the “ARE surveillance Lower Saxony”, a special project by the Governmental Institute of Public Health of Lower Saxony in cooperation with sentinel clinics and private practices throughout Lower Saxony to investigate causal pathogens for ARI in children [36]. The samples were tested for the following viruses: adenoviruses, influenza A and B viruses, human metapneumovirus, respiratory syncytial virus, and picornaviruses (including rhino- and enteroviruses). In case of multiple pathogens identified in the same sample, single viruses were weighted by 1/(n of pathogens) for further analyses.

The analysis of respiratory samples was primarily thought to be an incentive for participants; the aim of the study was not to provide pathogen-specific information on nasal swabs or stool samples, but to test if high-quality biomaterials can be taken.

### Perceptions regarding maintaining a symptom diary, obtainment of bio samples, and participation in a long-term study

At the end of the study, parents were asked about their perceptions regarding their acceptance of the study tasks, as well as other aspects of the long-term birth cohort study (participation, obtainment of blood) via questionnaire and telephone interviews.

### Statistical analyses

Feasibility was measured by means of return proportions of symptom diaries and bio samples. Reliability of the parental statements regarding visits to a pediatrician was tested against documentation at the doctors’ offices using Cohen’s kappa and classified according to the scale suggested by Landis and Koch (1977) [37]. A crude prevalence of symptoms was calculated as the number of days with symptoms in relation to the total number of days. Descriptive analyses were performed to analyze the acceptance of study tasks based on responses in the final questionnaire. We used multivariable logistic regression to detect predictive factors for compliance with the study protocol. Good compliance was defined as ≥75 days of the symptom diary filled and ≥3 nasal swabs and stool samples obtained. Variables that might influence compliance were chosen according to the literature (socioeconomic status indicated by education, age of mother at birth, age of child at study entry, child’s age at nursery school entry) and by a priori assumptions (number of siblings, behavior of child at first nasal swab). A backward elimination process (using p < 0.1 as the decision rule) was performed using the multivariable fractional polynomial approach proposed by Royston and Sauerbrei [38].

All analyses were performed with Stata for Windows, version 12 (StataCorp, College Station, TX).

### Human subjects review

The study protocol was approved by the Ethics Committee of Hannover Medical School and reviewed by the Federal Commissioner for Data Protection and Freedom of Information. Written informed consent was obtained from all participants.

## Results

### Sample

Out of 43 contacted nurseries, 38 with 899 children permitted the distribution of study flyers to the parents. The nurseries were well distributed across Braunschweig and included nurseries in different social areas. In 25 nurseries with a total of 612 children, the study was furthermore personally introduced to parents by study staff. Detailed study documents were handed out to 168 parents, of which 75 with children from 24 nurseries agreed to participate with their children in the study. The response proportion was 8.3 % based on all mailed flyers, or 12.2 % based on the number of children in nurseries where the study was presented.

At the beginning of the study, the children in the study sample (41 girls, 54.7 %) were between 13 and 38 months old (median: 25 months, interquartile range: 18–30). The parents in our study had a higher education than the German average [39], with more than half of them holding a master’s degree or doctorate (Table 1). When compared to the proportion of caesarian sections in Germany in the year 2010 [40] (31.9 %), a significantly higher proportion of children was born by caesarian section (47.1 %, 95 % CI: 35.1–59.4 %).

**Table 1:** Baseline characteristics of the study population

	Median (interquartile range) or number (%)
Mother’s age at birth (in years)	32 (29–34)
Mode of birth^a^	
Vaginal	37 (52.9)
Caesarian section	33 (47.1)
Child’s weight at birth (in g)^a^	3382 (2965–3752)
Child’s height at birth (in cm)^a^	52 (50–53)
Child’s age at nursery school entry (in months)^a^	14 (13–17)
Child’s age at study entry (in months)	25 (18–30)
Number of siblings^a^	
0	32 (50.8)
1	26 (41.3)
2	5 (7.9)
Highest education level of parents^a^	
- Low (uncompleted education)	0 (0)
- Middle (vocational training)	18 (26.5)
- High (higher vocational training or university degree)	50 (73.5)

^a^seven missing values for child’s weight and height at birth, education level of parents; five missing values for mode of birth; 13 missing values for number of siblings; 15 missing values for child’s age at nursery school entry

### Feasibility of symptom diary

The participants recorded the occurrence of their children’s symptoms for 5,584 days out of 6,750 (82.7 %). Fifty-eight participants (77.3 %) filled in 75 or more days of the symptom diary. Six participants did not provide any diary data. Of the 75 participants, 17 (22.7 %) chose to keep the symptom diary online. There was no difference between paper and online regarding the number of days entered (mean: 76.3 vs. 68.2, student’s t-test *p* = 0.315). The following problems with entries in the symptom diary occurred: (1) boxes ticked instead of providing numbers for strength/frequency of symptoms or instead of providing fever in °C, (2) unclear what to enter if none of the listed symptoms occurred but relevant other variables were to be ticked, and (3) unclear in which form and detail the medication should be entered.

Sixty-one participants (81.3 %) gave their consent to contact the child’s pediatrician. We received data from 13 out of 19 contacted pediatricians, covering 36 participating children. Five participants declared no visits to a doctor matching the doctor’s information. In 37 visits (50.7 %), there was a match between the registered visits. In 33 cases, a visit listed by the pediatrician was not stated by the parents. In most of these cases (*n* = 27, 81.8 %), the doctor’s visit was not due to respiratory or gastrointestinal symptoms. In eight cases, a visit declared by the parents was not confirmed by the pediatrician. Three visits stated by a pediatrician could not be verified due to a lack of symptom diary data. Overall, the agreement between parental records and doctor’s documentation was *substantial* (Cohen’s kappa = 0.62, 95 % CI: 0.52–0.72) or *almost perfect* after exclusion of visits which were not related to the symptoms or where diary data was missing (Cohen’s kappa = 0.82, 95 % CI: 0.73–0.91).

**Fig. 1:**
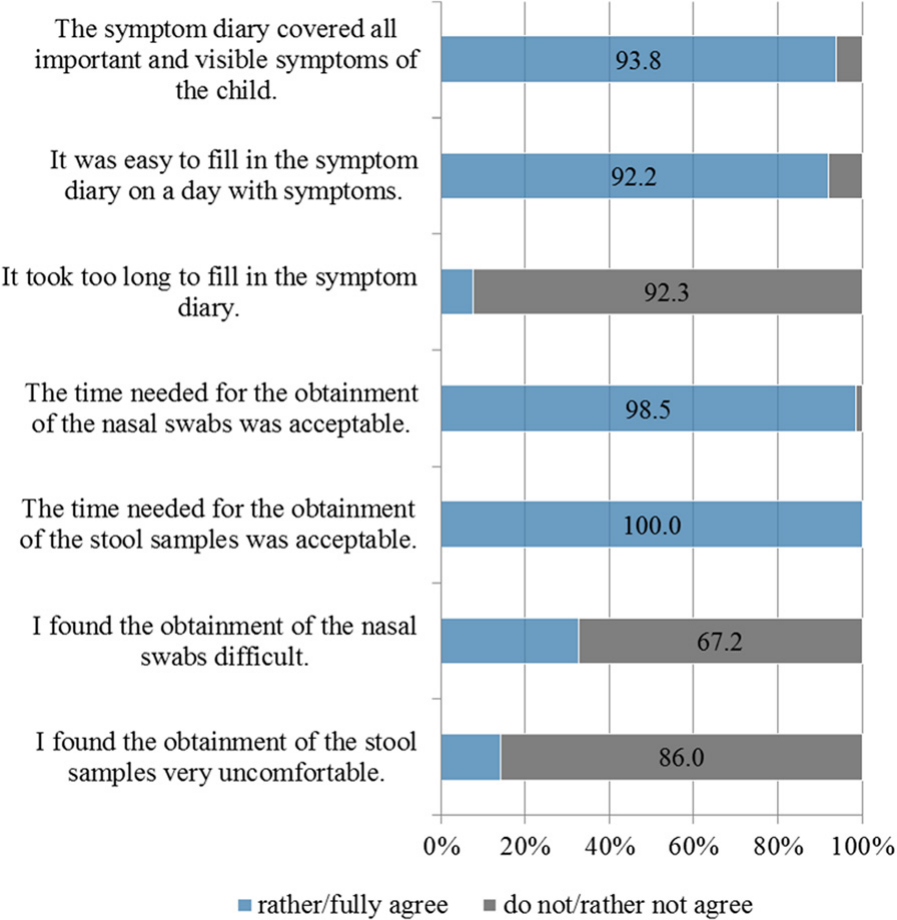
Acceptance of keeping the symptom diary and obtaining bio samples

Based on questionnaire responses, for 92.3 % of participants, filling in the symptom diary did not take too long (*n* = 65); 92.2 % agreed that entry on a day with symptoms was easy (*n* = 64, Fig. 1).

According to the question “When did you mainly fill in the symptom diary?”, half of the participants made their entries for several days (31, 48.4 %). About one third filled them in daily (19, 29.7 %), while the remaining 20 % can be further divided in those switching between “daily” and “for several days” (2, 3.1 %), those recording once a week (7, 10.9 %), and those entering data when symptoms occurred (5, 7.8 %).

### Occurrence of symptoms

On more than one third of the days with information registered, children suffered from a runny nose (37.9 %). The second most frequent symptom was coughing (25.9 %), followed by wheezing (3.9 %) (Table 2).

**Table 2:** Symptoms occurring during follow-up (*n* = 69)

Symptoms	Number of days	In % of all days filled in (5.584 days)
Runny nose	2117	37.9
Cough	1448	25.9
Wet cough	733	13.1
Dry cough	635	11.4
Cough (without specification)	80	1.4
Wheezing	215	3.9
Fever	212	3.8
Vomiting	41	0.7
Diarrhoea (≥3 times loose stool within 24 h)	38	0.7
Chills	27	0.5

### Feasibility of collection of bio samples

**Fig. 2:**
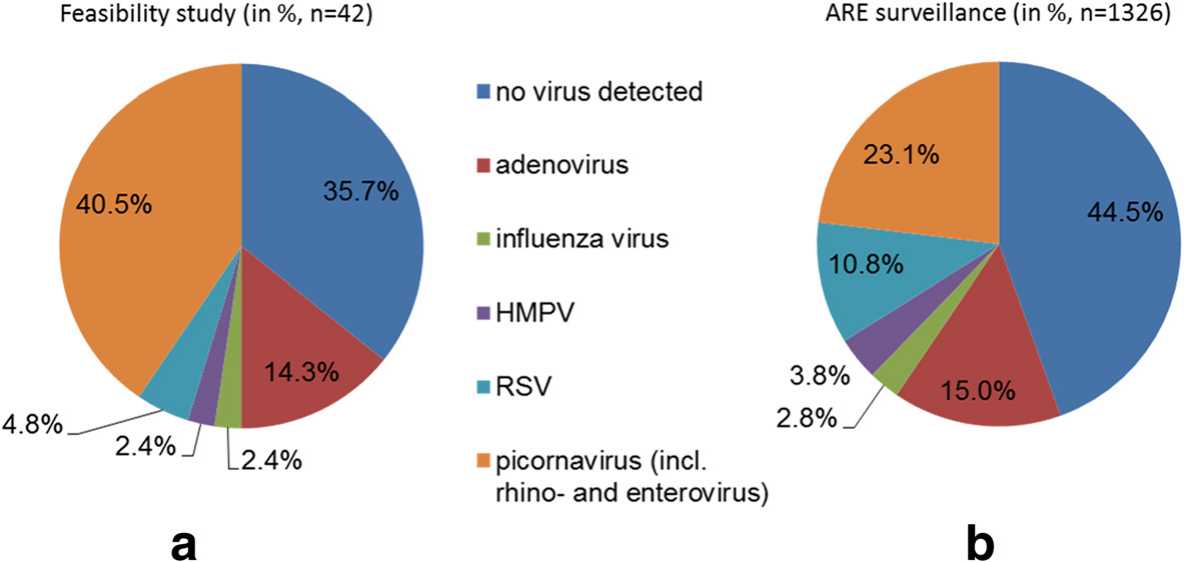
Virus findings (**a**) in the feasibility study and (**b**) in the ARE surveillance Lower Saxony, Germany. HMPV - human metapneumovirus, RSV - respiratory syncytial virus. In case of double or triple findings, the single virus results were counted as 0.5 respectively 0.333; dark blue: no virus detected; red: adenovirus; green: influenza virus; purple: HMPV; light blue: RSV; orange: picornavirus (incl. rhino- and enterovirus)

We received in total 230 nasal swabs and 205 stool samples. The majority of the participants provided three of the monthly nasal swabs (62.7 %) and stool samples (65.3 %). In 64.3 % of the symptom-triggered nasal swabs, respiratory viruses were detected. This was comparable to a 55.5 % virus detection proportion in the throat swabs obtained within the “ARE surveillance Lower Saxony” (two-sample test of proportions, *p* = 0.264, Fig. 2). The most frequently observed viruses in the 42 symptom-triggered nasal swabs were picornaviruses (40.5 %), followed by adenoviruses (14.3 %).

The median number of days between collecting the nasal swabs/stool samples and arrival at the study center was two days (interquartile range: 1–2 days).

Regarding the acceptance, 67.2 % of the participants reported collecting the nasal swabs as not being difficult, 70.3 % considered them not stressful for their child (Fig. 1). The most commonly occurring problems in obtaining the nasal swab were the child crying and fighting/struggling.

Most of the children tolerated the obtainment of nasal bio samples well. The mean value for the behavior of the children rated by the parents was 3.9 (standard deviation: 2.8, range: 1–10) based on all obtained swabs. For more than half of the nasal swabs (56.6 %), the behavior was rated between one and three; in 15.3 %, it was rated eight to ten. The mean value for the feelings of parents while obtaining the swab was 2.5 (standard deviation: 0.6, range: 0–3), 94.3 % felt confident or very confident.

86.0 % of participants found the obtainment of the stool sample not uncomfortable. The time needed to obtain the bio samples was acceptable for most people (nasal swabs: 98.5 %, stool samples: 100.0 %) (Fig. 1).

### Predictors of good compliance

In bivariate analyses, four variables were significantly associated with compliance (p < 0.25): mother’s age at birth, child’s age at nursery school entry, number of siblings, and parents’ education. In the multivariable analysis, a higher number of siblings was negatively associated with good compliance (OR = 0.3, 95 % CI: 0.1–1.0, *p* = 0.055), and higher education was positively associated with good compliance (OR = 1.5, 95 % CI: 1.1–2.2, *p* = 0.020).

### Outlook on longitudinal birth cohort study

About one third of the parents reported that they could imagine participation in a long-term study with daily symptom diaries (28.2 %) and frequent collection of stool samples (29.1 %) for at least 2 years, taking nasal swabs at symptoms (28.1 %) even up to their child’s school enrollment.

When assessing the feasibility of the long-term birth cohort study, we asked the participants in which form they wanted to fill in the symptom diary. The highest proportion of participants supported keeping the symptom diary on paper (82.5 %, *n* = 63), the second highest proportion wanted to use it as an app on a smartphone (58.3 %, *n* = 60), followed by online on a desktop PC or laptop (41.4 %, *n* = 58).

29.2 % of the participants would agree and 43.1 % would possibly agree to the obtainment of blood from their children within the scope of the study.

## Discussion

We were able to show in this study the general feasibility of a parent-administered data and sample collection approach which can potentially be used in future birth cohort studies aimed at investigating the longitudinal patterns of infection and immunity in early childhood. The use of a symptom diary and parental obtainment of bio samples can be beneficial to cohort studies investigating acute respiratory and gastrointestinal infections because it minimizes recall bias, which is a common problem in studies with retrospective data collection. Especially mild infections that resolve spontaneously and do not require the consultation of a physician tend to be forgotten. It is furthermore a resource-saving and low-threshold approach to identify pathogens compared to other approaches such as asking participants to visit their physician at every infection [19] or to send study staff to participants every week or every time participants report an infection [11, 20–23]. The approach tested in this study allows the investigation of a complete history of infections and can thus generate important evidence for pathogen-specific courses of disease and immune responses as well as for the holistic understanding of the interaction of different infections and their role for the development of immunity.

Within our study population, it was feasible for the majority of the participants to keep a symptom diary and to collect monthly nasal swabs and stool samples. The participants expressed good acceptance for the parental assessment of symptoms and collection of biomaterials. With 82.7 % of days registered, the completeness of the symptom diary was equal to or higher than that of previous studies with study periods of twelve (82.5 % [10]) and seven months (39 % [41]).

Due to problems occurring with entries in the symptom diary and the feedback we received from parents via interviews after the end of the study period, some adjustments of the symptom diary for the use in the birth cohort study seemed necessary: We now describe in more detail the form of the entries, emphasize when not to provide entries, and plan to check and discuss entries with the participants after one month of study participation. For use in the birth cohort, computer-assisted forms of the symptom diary might be an option to facilitate data collection, leading to avoidance of unspecific entries on paper and helping to save resources in the long run. Besides paper-based diaries, a relevant proportion of the participants supported keeping the symptom diary as a smartphone app. This might be an interesting and not yet researched opportunity for the assessment of symptom diary data.

As we only observed a three-month period during winter, comparable data regarding the number of days with symptoms or the ARI incidence are rare. However, runny/blocked noses and coughing are frequently shown to be the most often occurring respiratory symptoms [11, 41, 42].

The nasal swabs were suitable for identifying viral respiratory pathogens; the proportion of bio samples in which viruses were identified (64.3 %) lies in an acceptable range compared to studies with swabs taken by trained medical staff [11, 36]. The proportion of picornaviruses is much higher in the feasibility study than in the “ARE surveillance.” One potential explanation is that picornaviruses are usually associated with a less severe course of disease and our study population mainly experienced less serious infections compared to “ARE surveillance” where infections led to the consultation of a physician or a hospital stay. The participants were able to handle the additional burden of sending the bio samples via mail; overall, the bio samples arrived at the study center within two days after obtainment which, according to Lambert et al. [43], allows reliable virus identification if collection is not too long after the onset of symptoms.

The predictors for good compliance according to our model were the number of siblings and education. As our sample already consisted of people with above average education and our aim for the birth cohort study is to enroll a more representative study population, it is important to develop strategies to motivate participants of low or middle education levels to keep fulfilling the tasks. Continuous contact via e-mail, SMS, or phone might be an option, although studies with a clinical background showed conflicting results [44, 45]. While loss to follow-up didn’t play a role in this short-term study, it needs to be considered for sample size calculations for long-term birth cohort studies. As differential loss to follow-up might result in biased effect estimates, loss to follow-up needs to be minimized, and all efforts must be made to keep adherence high [46].

The population studied within this feasibility study was highly selective as, firstly, only 8–12 % agreed to participate and, secondly, the participants were highly educated. Therefore, the results cannot be generalized to the general population. While this is a huge issue when assessing measures of prevalence and incidence (e.g. with respect to pathogen detection), it is well described that a lack of external validity in cohort studies causes less problems since the focus of cohort studies is the investigation of associations within the study population. Effect estimates for these associations are usually unbiased in this case.

The cesarean section rate in our study population was considerably higher than in the German general population, showing again the selectiveness of the children observed. This will be important for the future birth cohort study as cesarean section has been shown to be associated with several measures of immunity as well as with the human microbiome [47].

Another limitation of our study is that we tested the practical feasibility for a period of three months only and, thus, cannot draw valid conclusions for the long-term compliance of the approach over several years. The choice to collect data during winter and spring was driven by the idea that this is the most difficult time for the parents to adhere to the study tasks as the burden of acute infections is highest. However, we cannot exclude that this might even have led to the opposite effect as parents were faced continuously with signs of infectious diseases and were therefore more likely to fill in the symptom diary and send back bio samples.

In order to get a better idea about the long-term compliance, we asked the participants about their attitude towards a longer participation. About one third of the participants could imagine maintaining a symptom diary and collecting stool samples at symptom occurrence for the first 2 years as well as symptomatic nasal swabs up to their child’s school enrollment. This implies that recruitment for a long-term study with these elements might be difficult, yet not impossible.

## Conclusions

We conclude that the parental assessment of symptoms and collection of biomaterials can be regarded as feasible in a middle class German population for the use in future community-based studies with the aim of investigating respiratory and gastrointestinal infections in children in Germany. The success of a long-term study will depend on the ability to maintain these activities over a long time period.

## Additional file

Additional file 1:
**Variables of the symptom diary and required form of entry.** (DOCX 31 kb)
