# Supplementary material for: Feasibility of a birth cohort study dedicated to assessing acute infections using symptom diaries and parental collection of biomaterials
Source: BMC Infect Dis. 2015 Oct 22;15:436. doi: 10.1186/s12879-015-1189-0 (PMC4618955; doi:10.1186/s12879-015-1189-0)
Supplement: Additional file 1: — Variables of the symptom diary and required form of entry. (DOCX 31 kb) [file 12879_2015_1189_MOESM1_ESM.docx]

Additional file 1: Variables of the symptom diary and required form of entry

| Variable | Form of entry |
| --- | --- |
| **General** |  |
| don’t know | to check, if parents cannot remember properly |
| no symptoms | to check, if child had no symptoms |
| **Symptoms** |  |
| fever | in Celsius degrees |
| body site of measurement | abbreviation from a provided list |
| wheezing | 1 (mild), 2 (middle) or 3 (strong) |
| cough - specified as dry, wet or without specification |  |
| runny nose |  |
| chills |  |
| loss of appetite |  |
| increased sleep requirement |  |
| increased clinginess |  |
| vomiting | number of vomiting episodes at particular day |
| loose stool | number of loose stools at particular day |
| **Impact Variables** |  |
| child absent from nursery | to check if applicable |
| parent absent from work because of child care | “M” for mother, “V” for father (in German), “MV” for both |

| parent absent from work because of infection | “M” for mother, “V” for father (in German), “MV” for both |
| --- | --- |
| visit to a pediatrician | to check if applicable |
| diagnosis of pediatrician | free entry |
| hospitalization | to check if applicable |
| antibiotics | to check if applicable |
| medication | name and duration of intake |
